# Deep Learning Model to Denoise Luminescence Images of Silicon Solar Cells

**DOI:** 10.1002/advs.202300206

**Published:** 2023-04-24

**Authors:** Grace Liu, Priya Dwivedi, Thorsten Trupke, Ziv Hameiri

**Affiliations:** ^1^ University of New South Wales (UNSW) Sydney NSW 2052 Australia

**Keywords:** denoising, luminescence imaging, machine learning, photovoltaics, U‐net model

## Abstract

Luminescence imaging is widely used to identify spatial defects and extract key electrical parameters of photovoltaic devices. To reliably identify defects, high‐quality images are desirable; however, acquiring such images implies a higher cost or lower throughput as they require better imaging systems or longer exposure times. This study proposes a deep learning‐based method to effectively diminish the noise in luminescence images, thereby enhancing their quality for inspection and analysis. The proposed method eliminates the requirement for extra hardware expenses or longer exposure times, making it a cost‐effective solution for image enhancement. This approach significantly improves image quality by >30% and >39% in terms of the peak signal‐to‐noise ratio and the structural similarity index, respectively, outperforming state‐of‐the‐art classical denoising algorithms.

## Graphical abstract

A state‐of‐the‐art U‐net model has been developed to effectively denoise luminescence images, resulting in a clearer image on the right from the noisy image on the left. The method outperforms conventional denoising techniques and has the potential to reduce solar cell production costs by increasing throughput and reducing the need for expensive imaging systems.

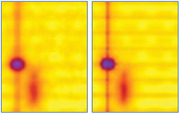

## 1 Introduction

In their 2022 assessment report on the impacts, adaptation, and vulnerability of climate change, the International Panel on Climate Change (IPCC) stated that a rise in global temperatures exceeding 1.5 °C will highly likely result in irreversible impacts on our planet.^[^ ^1^ ^]^ This high certainty calls for further development in policies and technologies that will mitigate climate change. Regarding photovoltaic (PV), the International Renewable Energy Agency (IRENA) states that the total solar PV installed capacity must reach almost 18 times the 2018 levels by 2050 in order to meet the Paris climate goals.^[^ ^2^ ^]^

PV is one of the most promising renewable energy technologies.^[^ ^3^ ^]^ The PV market has experienced significant growth in the last decade due to rapid technological advancements and substantial cost reduction.^[^ ^3^, ^4^ ^]^ However, further cost reduction is required to meet the Paris climate goals.^[^ ^2^ ^]^ This can be achieved by improving the efficiency and reliability of PV manufacturing through more advanced and cheaper inspection tools.^[^ ^4^ ^]^

Luminescence imaging is a key inspection method that is used for the characterization of crystalline silicon samples across the entire PV chain, from ingots to modules.^[^ ^5^, ^6^, ^7^, ^8^, ^9^, ^10^, ^11^ ^]^ Luminescence images are based on the emission of photons during radiative recombination.^[^ ^12^ ^]^ Electroluminescence (EL)^[^ ^13^ ^]^ is induced by injecting an electrical current into fully processed solar cells, while illumination with a light source is used to produce photoluminescence (PL).^[^ ^12^ ^]^ The luminescence emission is captured by an infrared camera, and the resulting images reveal solar cell defects and faults that are usually undetectable to the naked eye.^[^ ^14^ ^]^ Luminescence imaging has also been used to predict the electrical performance of solar cells.^[^ ^14^, ^15^, ^16^ ^]^ High luminescence image quality is generally desirable for both quantitative and qualitative analysis methods, enabling more reliable and accurate analysis. A particular challenge in achieving high‐quality images is reducing noise. It is widely recognized that image noise reduction is a crucial aspect of luminescence image inspections, especially for outdoor imaging, which tends to be exposed to higher levels of noise.^[^ ^17^ ^]^

Several classical algorithms exist to remove noise.^[^ ^18^ ^]^ Two of the most popular algorithms include block‐matching and 3D filtering (BM3D),^[^ ^19^ ^]^ and weighted nuclear norm minimization (WNNM).^[^ ^20^ ^]^ BM3D is an extension of the non‐local means algorithm.^[^ ^21^ ^]^ It groups image patches of similar intensities, removes their noise, and restores them to the original image. WNNM improves the nuclear norm minimization algorithm^[^ ^22^ ^]^ by assigning weights to singular values that produce a more effective low‐rank matrix; once found, it denoises them. In general, WNNM often outperforms BM3D by a small margin, but WNNM is computationally more expensive.^[^ ^18^, ^23^ ^]^ Recently, however, these classical denoising algorithms have been losing popularity with the rise of deep learning, which often has superior performance.^[^ ^18^, ^23^, ^24^, ^25^ ^]^

Deep learning,^[^ ^26^ ^]^ which involves the use of neural networks,^[^ ^27^ ^]^ is a type of machine learning algorithm that has seen incredible applications in a wide range of areas over recent years^[^ ^28^ ^]^ including medical classification,^[^ ^29^ ^]^ speech recognition,^[^ ^30^ ^]^ and many more.^[^ ^16^, ^24^ ^]^ Deep learning has also been of interest to the PV community^[^ ^31^ ^]^ including within PV fault classification^[^ ^32^ ^]^ and irradiance forecasting.^[^ ^33^ ^]^ Its promise lies in the flexibility of its design which gives it an incredible ability to adapt to diverse problems given a suitable dataset, compared to single‐purpose rules and formulas designed for specific problems such as classical denoising algorithms. The models that have shown high performance in denoising applications^[^ ^24^ ^]^ include autoencoders,^[^ ^34^ ^]^ generative adversarial networks (GANs),^[^ ^35^ ^]^ and U‐nets^[^ ^36^ ^]^ with GANs and U‐nets performing particularly well.^[^ ^37^ ^]^ Relevant to this study, Kurumundayil et al. have successfully used a GAN to improve the image quality of blurred EL images^[^ ^38^ ^]^ and to remove unwanted marks when analyzing silicon wafer images.^[^ ^39^ ^]^

This paper investigates the efficacy of using U‐net, a deep learning model, to reduce noise in both EL and PL images. Previous research in other fields has demonstrated that U‐nets outperform other models, including GANs, in terms of its architectural simplicity and effectiveness in denoising.^[^ ^37^, ^40^, ^41^ ^]^ This study presents, for the first time, the effectiveness of deep learning in general, and U‐net in particular, for denoising luminescence images.

## 2 Experimental Section

Two datasets of nine‐busbar monocrystalline solar cells were used in this study: 9600 pairs of noisy and clean EL images (520 × 520 pixels; 7680 for training and 1920 for testing) and 3120 pairs of noisy and clean PL images (1024 × 1024 pixels; 3000 for training and 120 for testing). The EL images were used in the initial development and testing of the U‐net model, while the PL images were used for fine‐tuning the model and experimental validation.^[^ ^42^ ^]^

The EL dataset was created from clean EL images that were captured by an industrial EL imaging system, while the matching noisy EL images were generated by artificially noising their clean versions with Gaussian noise^[^ ^43^ ^]^ (mean of zero and random variance between 0.0001 and 0.001) and Poisson noise^[^ ^44^ ^]^ (10^6^ photons that incident on the camera sensor). Similarly, the PL dataset was created by noising clean PL images that were taken by a commercial PL system. Images that were used for training were image patches (random cropped) which improved the generalization of the trained U‐net model.

**Figure 1:**
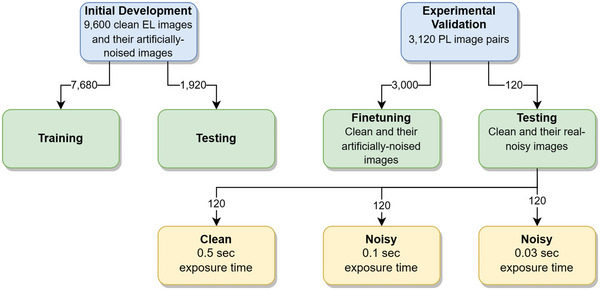
Dataset summary.

The experimental validation test dataset consisted of 120 PL images taken with an exposure time of 0.5 s (“clean” images). The noisy images were taken with exposure times of 0.1 s (120 images) and 0.03 s (120 images). As expected, the noise in the image increases when the exposure time decreases (see below). **Figure** **1** provides a summary of the datasets described.

**Figure 2:**
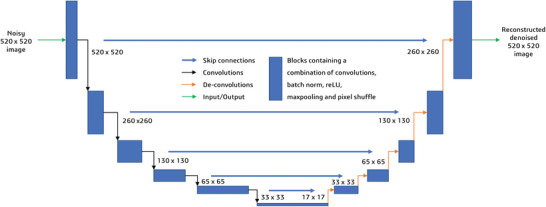
FastAI Resnet‐34 U‐net.

Differing from the original U‐net architecture,^[^ ^36^ ^]^ a Resnet‐34^[^ ^45^ ^]^ was specially developed as the U‐net's encoder pre‐trained on the ImageNet dataset.^[^ ^46^ ^]^ Pre‐training has been widely successful in improving computer vision models as it provides the models with the benefits of the visual object recognition fundamentals without the need for large amounts of resources (computation, time, etc.) that would otherwise be required.^[^ ^47^ ^]^ Additionally, the connections between the input and the output were optimized by additional skip connections and the use of pixel shuffle.^[^ ^48^ ^]^ These were implemented using the FastAI library. **Figure** **2** presents the developed U‐net architecture used in this study. The model was trained using an Adam optimizer with a learning rate of 0.001,^[^ ^49^ ^]^ a mean square error loss function,^[^ ^50^ ^]^ and a batch size of four.

The performance of the developed deep learning model was then compared to the traditional denoising BM3D algorithm.^[^ ^19^ ^]^ BM3D was chosen over WNNM due to its lower computational cost and competitive performance,^[^ ^51^ ^]^ and as it is often used as a benchmark.^[^ ^18^, ^52^ ^]^ To assess the performance of the BM3D and the deep learning models, the noisy and denoised images were compared to their corresponding original clean images through three evaluation metrics: the peak signal‐to‐noise ratio (PSNR),^[^ ^53^ ^]^ the structural similarity index metric (SSIM),^[^ ^53^, ^54^ ^]^ and visual inspection.

The PSNR quantifies (in dB) the differences between pixel values in two images. It ranges from zero to infinity with a smaller value implying lower image quality:

(1) $$\mathrm{PSNR}=20\log_{10}\frac{L}{\sqrt{\mathrm{MSE}}}$$

Here *L* is the maximum possible pixel value of the image and MSE is defined as:

(2) $$\mathrm{MSE}=\frac{1}{N}\sum_{i=1}^{N}{\left(y_{i}-{\hat{y}}_{i}\right)}^{2}$$

where *N* is the number of training examples, *y*_i_ is the original value, and ${\hat{y}}_{i}$ is the predicted value by the model.

The SSIM measures the structural similarity in the visual features between two images. It ranges between zero and unity. Zero indicates no structural similarity while unity indicates a perfect similarity:^[^ ^53^ ^]^

(3) $$\mathrm{SSIM}=\frac{\left(2\mu_{x}\mu_{y}+C_{1}\right)\left(2\sigma_{xy}+C_{2}\right)}{\left(\mu_{x}^{2}+\mu_{y}^{2}+C_{1}\right)\left(\sigma_{x}^{2}+\sigma_{y}^{2}+C_{2}\right)}$$

where *x* and *y* are the pixel values from two images being compared, *μ* is the mean, $\sigma_{i}^{2}$ is the variance of *i*, *σ*_*ij*_ is the covariance of *i* and *j*, and *C_i_* is a constant included to avoid instability when $\mu_{x}^{2}+\;\mu_{y}^{2}$ and $\sigma_{x}^{2}+\;\sigma_{y}^{2}\;$are close to zero.

Although the PSNR evaluated the quality of images, it cannot account for structural similarities between images which are a key component in human vision.^[^ ^54^, ^55^, ^56^ ^]^ Hence, the PSNR and SSIM were often used together. Nevertheless, visual inspection was essential to confirm the obtained results regardless of the used metrics.^[^ ^18^, ^24^, ^25^, ^57^ ^]^

## 3 Results and Discussion

**Figure 3:**
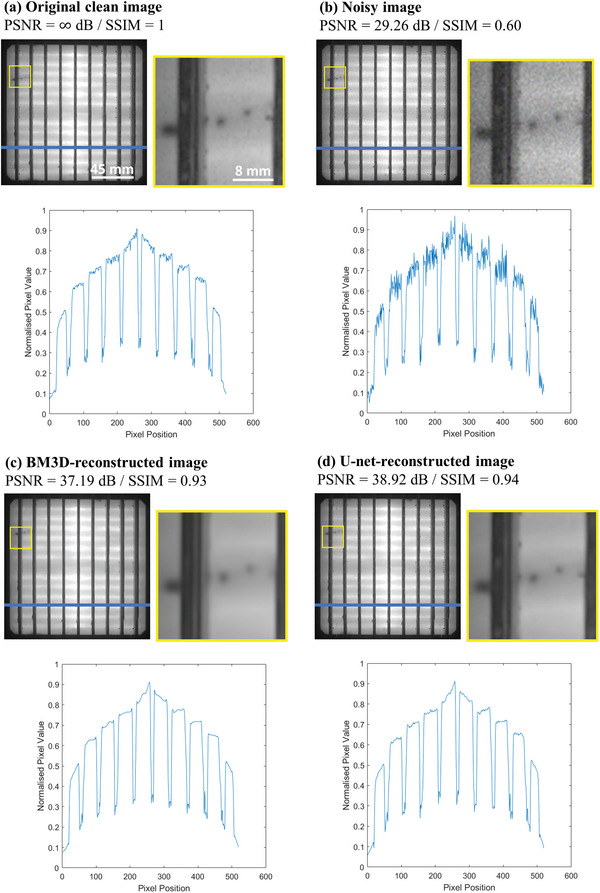
Denoising of the EL image: a) a representative original EL image, b) a noisy image, c) a BM3D‐reconstructed image, and d) a U‐net‐reconstructed image. Corresponding PSNR and SSIM are included for quantitative comparison.

**Figure** **3** presents a representative pair of clean (a) and noisy (b) EL images from the test set. The figure also includes the BM3D‐reconstructed (c) and U‐net‐reconstructed (d) images. Representative zoomed‐in regions are incorporated to assist with the visual inspection. To further highlight any noise difference, the pixel profiles along the blue lines (drawn across each EL image) are plotted.

Referring to the zoomed‐in regions, it can be observed that the noisy image has a significantly grainier appearance compared to the original clean image. This is further highlighted by the reduction in both the PSNR and SSIM (≈29.26 dB and ≈0.60, respectively) and by its noisier profile graph. The reconstruction process dramatically reduces the noise as the zoomed‐in regions and profile graphs appear to have little to no noise, and the PSNR and SSIM of the reconstructed images are significantly higher than those of the noisy image. It is noted that the profile graphs of the reconstructed images have retained the shape of the original image profile graph. Though both reconstructed images retain the main features and are successfully denoised, it is noticeable that the U‐net reconstruction outperforms the BM3D algorithm with a higher PSNR and SSIM. Furthermore, the U‐net model reconstructs all the major and minor features of the original image, such as the busbars, background luminescence, small defects, and measurement contacts, whereas the BM3D‐reconstructed image is missing some subtle features (such as the details at the busbars). It also seems that the defect in the zoomed‐in section is slightly blurred in the BM3D‐reconstructed image, while in the U‐net's reconstruction; it is nearly indistinguishable from the original image. Interestingly, with a closer comparison, it even seems that the reconstructed images look less noisy than the original image.

**Figure 4:**
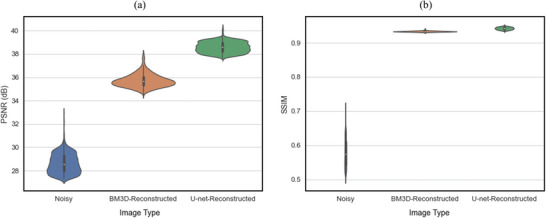
Results of the test set showing the a) PSNR and b) SSIM violinplots of the noisy, BM3D‐reconstructed, and U‐net‐reconstructed images of the test set. Each distribution has its mean, and first, second, third, and fourth quartiles marked in the vertical bar.

**Figure** **4** presents the PSNR (a) and SSIM (b) distribution of the noisy, BM3D‐reconstructed, and U‐net‐reconstructed images of the test dataset (1920 unseen EL images). The noisy images averaged a PSNR of 28.64 dB and an SSIM of 0.58. Both of the reconstruction methods significantly improve the quality of the images with the U‐net overperforming with much higher PSNR (38.59 dB vs 35.74 dB) and similar SSIM (0.94 vs 0.93) to the BM3D results. Note that PSNR is in logarithmic scale; therefore, a small variation in PSNR corresponds to a large variation in pixel‐wise loss. Interestingly, the BM3D algorithm seems more consistent with its SSIM results as it has a narrower distribution than the U‐net.

**Figure 5:**
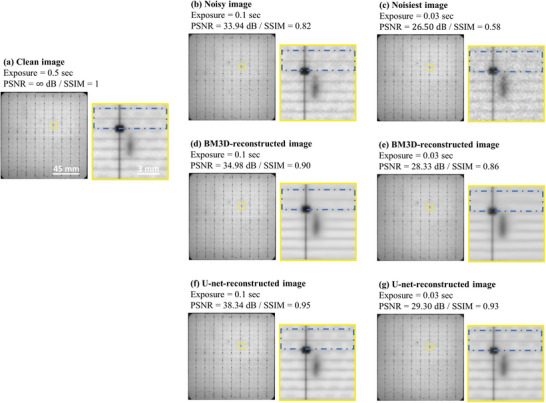
Denoising of the PL images: a) a representative original image, noisy images taken with an exposure time of b) 0.1 s and c) 0.03 s; denoising of the d) 0.1 s and e) 0.03 s exposure time image using BM3D; and denoising of the f) 0.1 s and g) 0.03 s exposure time image using U‐net. Corresponding PSNR and SSIM are shown for quantitative comparison.

As the model was developed on a dataset that used synthetic noise, experimental validation on real‐noisy images is necessary. **Figure** **5** shows representative images from the experimental validation set: a) a clean image taken with an exposure time of 0.5 s; the noisy versions of this image were taken with exposure times of 0.1 s (b) and 0.03 s (c). A lower exposure time results in a noisier image. This is reflected visually through a higher graininess that makes details harder to observe and reflected quantitatively through a lower PSNR and SSIM. For example, in (c), which was taken with an exposure time of 0.03 s, the fingers (see within the blue dotted box) are barely visible and have the lowest evaluation metrics compared to (a) and (b) which were taken at an exposure time of 0.5 and 0.1 s, respectively.

The BM3D‐reconstructed (d, e) and the U‐net‐reconstructed (f, g) images demonstrate a strong resemblance to the clean original image with all key features retained such as the busbars and defects. There is a clear improvement in the PSNR and SSIM, and visually, the noise is removed. Both denoising algorithms face challenges when the noise is higher, as evidenced by the lower quality of the reconstructed images. Both can only slightly improve the PSNR of the images taken with 0.03 s exposure time compared to their reconstruction of the 0.1 s images. However, although the evaluation metrics are low for the 0.03 s images, visually, the U‐net has been able to retain the minor details of the original, such as the fingers (marked with the blue dotted box). This is particularly interesting as the fingers are very difficult to spot in the 0.03 s exposure time noisy image.

**Figure 6:**
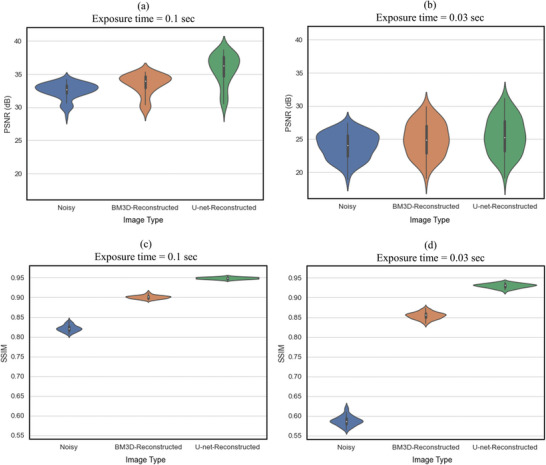
Results of the test set showing the a,b) PSNR and c,d) SSIM violinplots of the U‐net‐reconstructed and BM3D‐reconstructed image for the noisy image taken with the exposure times of a,c) 0.1 and b,d) 0.03 s. Each distribution has its mean, and first, second, third, and fourth quartiles are in the vertical bar.

These statistical results for the entire PL image set are shown in **Figure** **6** which summarizes the PSNR and SSIM of the validation test (120 images). Compared to the SSIM distributions which are separate and distinct, there is an overlapping distribution of the PSNR between the noisy and reconstructed images. This differs from Figure 4, indicating that the image structure improved greatly but a large number of individual pixels are different between the original and reconstructed images. One possible explanation for this discrepancy is that the different exposure times produced slightly different PL images in terms of different light intensity distributions. This is a challenge as the denoising algorithms assume that only the amount of noise changes with different exposure times. However, this issue can be addressed by extending the training dataset to include more PL images taken with different exposure times. Note that this adjustment can only be applied with the U‐net whereas the BM3D algorithm would not be able to easily adapt to this discrepancy in the light intensity distribution. Nevertheless, as discussed, both PSNR and SSIM need to be evaluated together to assess image quality.^[^ ^58^ ^]^ The significant improvement in the SSIM seems to result in a notable improvement in the image quality as confirmed by the visual inspection. Overall, the U‐net appears to perform a successful reconstruction. The reduction in the exposure time from 0.5 to 0.03 s can lead to 16 times faster imaging throughput; a significant enhancement for existing and new imaging systems (such as those that are used for outdoor imaging).

As an additional note, when evaluating the experimental validation results, it is important to emphasize that the U‐net model was developed using EL images and was only finetuned with the PL images. Remarkably, the EL‐based model could be adapted, by just tuning its pre‐trained parameters, for an entirely different type of luminescence image acquired with different hardware and in a different image size.

## 4 Conclusion

We presented a novel deep‐learning U‐net application for denoising and thereby significantly improving the quality of luminescence images of silicon solar cells. The developed U‐net model outperforms the classical BM3D denoising algorithm with an increase of >30% in the PSNR metric and >39% in the SSIM. Its success is visually reflected in the reconstructed images, which are nearly indistinguishable from the original images. These results highlight the significant potential of the proposed approach in enabling luminescence image data with reduced noise, without the need for long exposure times (for example, in outdoor photoluminescence imaging) or expensive luminescence imaging hardware. Furthermore, due to the flexible nature of deep learning models (as demonstrated by its ability to easily adapt to both EL and PL images), the developed method has the potential to be extended to other types of PV‐related images such as infrared, optical, and hyperspectral images.

## Conflict of Interest

The authors declare no conflict of interest.

## Data Availability

The data that support the findings of this study are available from the corresponding author upon reasonable request.
